# Successful Modification of a Commercial Wheat Variety, Lunxuan 13, for Pre-Harvest Sprouting Resistance Through Editing of the *TaQsd1* Gene

**DOI:** 10.3390/plants15091322

**Published:** 2026-04-25

**Authors:** Zhiyang Han, Liqiang Yu, Xi Li, Surong Wang, Ke Ding, Buquan Zhao, Weihong Huang, Hao Peng, Yang Zhou, Ke Wang, Huali Tang, Xingguo Ye

**Affiliations:** 1 State Key Laboratory of Crop Gene Resources and Breeding, National Key Facility for Crop Gene Resources and Genetic Improvement, Institute of Crop Science, Chinese Academy of Agricultural Sciences, Beijing 100081, Chinawangke03@caas.cn (K.W.); 2 Key Laboratory of Molecular Epigenetics of Ministry of Education, Northeast Normal University, Changchun 130024, China; 3 Zhaoxian Experiment Station of Shijiazhuang Academy of Agriculture and Forestry Research, Shijiazhuang 051530, China

**Keywords:** Lunxuan 13, wheat, pre-harvest sprouting, CRISPR/Cas9, *TaQsd1*, seed dormancy, gene editing

## Abstract

Wheat is a globally important food crop, and its yield is crucial for ensuring food security. Lunxuan 13 is an elite wheat variety developed by the Institute of Crop Sciences, Chinese Academy of Agricultural Sciences. It has high yield potential and outstanding agronomic traits, such as excellent seed setting rate, plump kernels, and good lodging resistance. However, this variety is highly susceptible to pre-harvest sprouting (PHS) when exposed to rain during the maturation period, leading to premature grain germination on the spike, which causes yield losses and quality deterioration, severely restricting its popularization. This study focused on addressing the PHS susceptibility of Lunxuan 13 by employing CRISPR/Cas9 technology for the targeted knockout of the three homoeologous copies (A, B, and D subgenomes) of *TaQsd1*, a key gene regulating seed dormancy. A total of 41 transgenic plants were obtained, achieving a transformation efficiency of 52.6%, among which 27 plants exhibited edits at the target sites, resulting in an editing efficiency of 65.9%. Phenotypic analysis of homozygous T_2_ edited lines revealed significant functional redundancy among the three *TaQsd1* homoeologs: a significant extension of the seed dormancy period and a substantial increase in PHS resistance were achieved only when all three A, B, and D copies underwent loss-of-function mutation (*aabbdd* genotype). After-ripened seeds from these mutants showed normal germination ability, indicating enhanced dormancy rather than loss of germination capacity. Importantly, all of the edited lines exhibited no significant differences compared to the wild type in key agronomic traits such as plant height, spike length, and grains per spike, thus retaining the excellent characteristics of Lunxuan 13. This study successfully optimized Lunxuan 13 for significantly enhanced PHS resistance while retaining its superior agronomic traits. This work provides an effective approach for improving PHS resistance in white-grained wheat and removes a key barrier to the potential commercialization of this variety.

## 1. Introduction

As one of the world’s most vital cereal crops, common wheat (*Triticum aestivum* L.), with its remarkable adaptability and high yield potential, serves as a primary dietary energy source for over 35% of the global population, playing an indispensable role in ensuring worldwide food security [1]. However, under the dual pressures of continuous global population growth and intensifying climate change, breaking the yield ceiling of wheat to meet escalating food demands has become a critical challenge in agricultural research [2]. Wheat yield is determined by multiple factors, including productive tillers per unit area, grains per spike, thousand-kernel weight, spike uniformity, and stress resistance. Among these, three components are particularly crucial: productive tillers per unit area, grains per spike, and thousand-kernel weight. With the ongoing advancement of global agricultural modernization, the potential for further increasing tiller density has become increasingly constrained, while thousand-kernel weight remains largely limited by the genetic characteristics of cultivars. Under these circumstances, in-depth investigation of the regulatory mechanisms governing grain number per spike and optimization of its key determinants has emerged as a pivotal research focus for overcoming current yield limitations in wheat production [3].

Lunxuan 13 is an elite white-grained wheat cultivar developed by the Institute of Crop Sciences, Chinese Academy of Agricultural Sciences. It was released in 2018 and is suitable for planting in the northern winter wheat regions of China, including Beijing, Tianjin, and Hebei Province. As a facultative variety with a growth period of approximately 240 days, it exhibits superior agronomic traits including excellent seed setting rate, plump kernels, and remarkable yield potential. Field evaluations have demonstrated its outstanding performance, with average yields consistently exceeding control varieties by more than 5% in national regional trials during 2013–2015, reaching up to 8.2 t/ha [4]. However, this variety exhibits a significant drawback: it is prone to PHS when exposed to prolonged rain or high humidity during the ripening stage. The premature germination of grains on the spikes before harvest not only reduces yield but also severely impairs processing quality, potentially leading to substantial economic losses in agricultural production [5,6].

PHS refers to the premature germination of cereal grains on the spike before or during harvest due to prolonged wet or high-humidity conditions [7,8,9]. In the major wheat-producing regions of China, such as the Huang–Huai–Hai Plain, seasonal rainfall during the ripening period occurs frequently, leading to significant PHS outbreaks and severe yield losses annually [10]. During sprouting, increased activity of amylases and proteases degrades stored starch and proteins, lowering test weight and thousand-kernel weight, thereby reducing yield. Additionally, the breakdown of gluten proteins impairs flour processing quality, leading to reduced dough viscosity, poor baking performance, and downgrading to feed-grade use in severe cases. Moreover, sprouted grains are prone to mold contamination, increasing the risk of mycotoxins such as deoxynivalenol (DON) and aflatoxins, posing food safety hazards [10,11,12,13].

Research has demonstrated a strong correlation between seed dormancy characteristics and PHS resistance [14]. Seeds with low dormancy are more prone to PHS, while excessive dormancy may negatively impact germination uniformity and subsequent seedling establishment. Notably, modern wheat cultivars commonly exhibit weak dormancy and poor PHS resistance [15]. Therefore, appropriately enhancing seed dormancy through genetic improvement can effectively mitigate the adverse effects of PHS on yield and grain quality [15,16,17]. Previous studies demonstrated an association between the barley seed dormancy gene *Qsd1* (quantitative trait locus on seed dormancy 1) and PHS tolerance, where loss of *Qsd1* function leads to prolonged seed dormancy [18]. Given the high genomic homology between barley and wheat, a novel wheat germplasm with stable PHS resistance was developed by employing gene-editing technology to target and knock out the *Qsd1* ortholog in wheat [19].

Gene editing is a revolutionary approach that enables precise modification of endogenous genes in organisms using nucleases. Operations such as targeted knock-in or knock-out have demonstrated tremendous potential in both gene function research and gene therapy [20]. Among the genome editing technologies developed recently, the CRISPR/Cas9 system has emerged as a core tool for crop genetic improvement and trait enhancement due to its advantages of simple design, high editing efficiency, and low cost [21]. In plant genome editing, the CRISPR/Cas9 components are typically delivered via biolistic bombardment or *Agrobacterium*-mediated transformation, integrating exogenous DNA (containing the Cas9 expression cassette and sgRNA expression elements) into the plant genome. Subsequently, the Cas9 nuclease, guided by the sgRNA, induces site-specific cleavage at the target locus, leading to gene mutations through either non-homologous end joining (NHEJ) or homology-directed repair (HDR) pathways [22,23,24]. In recent years, with breakthroughs in efficient genetic transformation systems for wheat, the application of *Agrobacterium*-mediated CRISPR/Cas9 systems in wheat genome editing has become increasingly widespread [25,26,27]. By using the editing system, many improvements in important agronomic traits have been achieved in wheat [28,29,30,31].

The primary objective of this research was to improve the PHS susceptibility of the high-yielding wheat variety Lunxuan 13 through targeted genetic modification. We therefore employed an optimized *Agrobacterium*-mediated CRISPR/Cas9 system to perform genome editing on the *Qsd1* homologous loci in the wheat cultivar Lunxuan 13. Three mutant lines with edited homologs were successfully obtained, and they exhibited significantly stronger PHS resistance compared to the wild-type Lunxuan 13. The new lines might be potentially used in wheat production in the future after further trial tests and biosafety evaluation by replacing Lunxuan 13.

## 2. Results

### 2.1. Knockout of Three TaQsd1 Allele Genes in Wheat via CRISPR/Cas9 Technology

**Figure 1:**
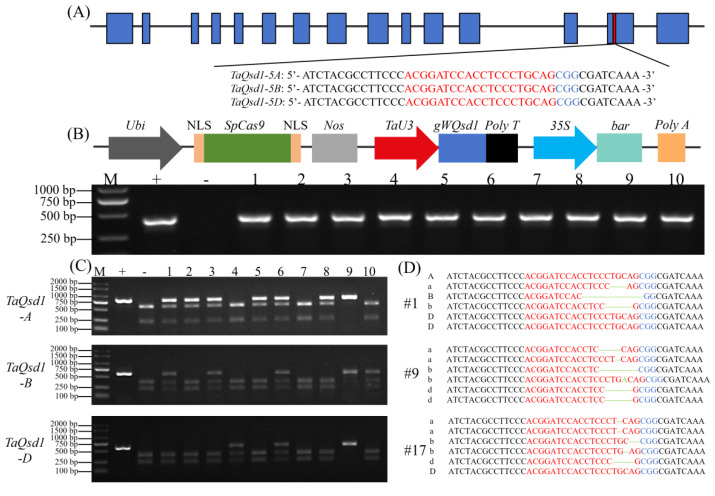
Targeted knockout of *TaQsd1* via *Agrobacterium*-mediated transformation using CRISPR/Cas9. (**A**) Linearized expression vector structure of *pWMB110-Cas9-TaQsd1*; blue letters indicate the PAM sequences; red letters indicate the sgRNA sequences. *35S*, *CaMV35S* promoter; *bar*, phosphinothricin resistance gene; NLS, nuclear localisation signal; *Nos*, Nos terminator; *TaU3*, the wheat U3 promoter; *Ubi*, ubiquitin promoter. (**B**) PCR amplification of *bar* in T_0_ plants; M, marker; +, vector *pWMB110-Cas9-TaQsd1*; -, wild-type Lunxuan 13; 1–10, T_0_ plants. (**C**) PCR/RE detection of *TaQsd1* edits in T_0_ transgenic plants; M: DNA marker; +: Undigested PCR product; -: Digested wild-type control; 1–10: RE-digested T_0_ samples. (**D**) Nucleotide sequences showing genome editing at the *TaQsd1* locus. The target sequence and PAM are indicated as in (**A**). Deleted and inserted nucleotides are shown in green.

To perform genome editing on the homologous alleles of the *TaQsd1* gene in wheat cultivar Lunxuan 13, we designed a 20 bp guide RNA (gRNA) targeting a conserved region within the exons of the three *TaQsd1* alleles, which can simultaneously target the A, B, and D subgenomes and contains a restriction enzyme recognition site located 3 bp upstream of the protospacer adjacent motif (PAM) (Figure 1A). For editing the *TaQsd1* homologous genes, a total of 78 immature embryos from Lunxuan 13 were transformed by the *Agrobacterium* cells harboring a vector *pWMB110-Cas9-TaQsd1* co-expressing *Cas9* and the *gRNA*. After preliminary screening by means of PCR detection using *bar* gene-specific primers, 41 T_0_ transgenic plants were obtained, with a transformation efficiency of 52.6% (Figure 1B and Table 1).

Further validation by means of amplification with *TaQsd1*-specific primers and *Pst*I restriction enzyme digestion (PCR/RE) identified 27 T_0_ plants with editing events at the target site, yielding an editing efficiency of 65.9% (Table 1). Among these plants, 23 were heterozygous and 3 were homozygous mutants. Specifically, among the identified T_0_ edited plants, mutations in the *TaQsd1-5A*, *TaQsd1-5B*, and *TaQsd1-5D* homologous genes were detected in 21, 23, and 17 plants, corresponding to editing efficiencies of 51.2%, 56.1%, and 41.5% in order (Table 1). Regarding mutation types, 7 plants had mutations in only one of the three homologous genes, accounting for 25.9% in the edited plants (Table 1); 6 plants had simultaneous mutations at two loci, accounting for 22.2% (Table 1); and another 14 plants exhibited mutations in all three alleles, accounting for 51.9% (Figure 1C and Table 1). Sanger sequencing revealed that the primary knockout types for *TaQsd1* in the edited plants included deletions of 1–11 bp at the 3 bp upstream of the PAM site within the sgRNA, and a 1 bp insertion was also identified (Figure 1D). The mutation genotypes at the loci in this generation involved *aabbDD*, *Aabbdd*, *AAbbDD*, *AabbDD*, *aabbdd*, *AABbDD*, *aabbDd*, *AABbDd*, and *AABbdd*. These results indicate that the CRISPR/Cas9 system performed effectively for editing *TaQsd1* genes in Lunxuan 13.

### 2.2. Off-Target Validation

To ensure the specificity of the CRISPR/Cas9 system in editing wheat *TaQsd1* genes, we screened the wheat reference genome for the *TaQsd1* sgRNA sequence and identified three potential off-target genomic sites for validation analysis. These sites contained three or five nucleotide mismatches compared to the target sequence of *TaQsd1*. Detection by means of PCR-RE combining Sanger sequencing on T_1_ edited plants showed that no editing mutations occurred at any of the three potential off-target sites (Supplementary Table S2). This result indicates that, under our experimental conditions, CRISPR/Cas9 editing of *TaQsd1* was highly specific, and off-target effects could be effectively excluded.

### 2.3. Genetic Inheritance Detection in the T_2_ Generation

**Figure 2:**
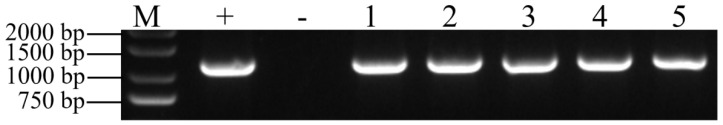
PCR amplification of *Cas9* in T_1_ plants; M, marker; +, vector *pWMB110-Cas9-TaQsd1*; -, wild-type Lunxuan 13; 1–5, T_1_ plants.

To verify the genetic stability of the *TaQsd1*-edited plants and the sustained activity of *Cas9* in the T_2_ generation, we randomly selected five T_1_ edited plants carrying the *Cas9* expression cassette (Ya33-1-3, Ya33-1-6, Ya33-6-2, Ya33-17-4, and Ya33-25-7) and produced T_2_ edited plants for analysis (Figure 2).

Editing detection results showed that at the *TaQsd1-5A* sgRNA target site, Ya33-1-6 and Ya33-17-4 harbored a 1 bp deletion at the position 3 bp upstream of the PAM. At the *TaQsd1-5B* sgRNA target site, Ya33-1-6, Ya33-6-2, Ya33-17-4, and Ya33-25-7 exhibited either a 3 bp or an 11 bp deletion at the position 3 bp upstream of the PAM. At the *TaQsd1-5D* sgRNA target site, a 1 bp insertion was detected at the position 3 bp upstream of the PAM in Ya33-1-3, Ya33-17-4, and Ya33-25-7. Genotype analysis indicated that Ya33-6-2 and Ya33-17-4 were homozygous knockout lines, while Ya33-1-3, Ya33-1-6, and Ya33-25-7 were heterozygous knockout lines in the T_1_ generation (Table 2). Further Sanger sequencing of 20 T_2_ plants derived from the aforementioned five T_1_ lines revealed that the editing types of *TaQsd1* in the T_2_ generation were completely consistent with those in the T_1_ generation, without new mutation types generated. The segregation ratios of biallelic mutations, heterozygous mutations, and wild type among the progeny in each line followed Mendelian inheritance patterns (approximately 1:2:1) (Table 2). These results indicate that genome editing in wheat occurred only during the transformation and regeneration period, and no new mutations or phenotypic changes were induced in the T_2_ generation despite the persistent presence of *Cas9* and sgRNA.

### 2.4. Dormancy Period Determination and Germination Assay

**Figure 3:**
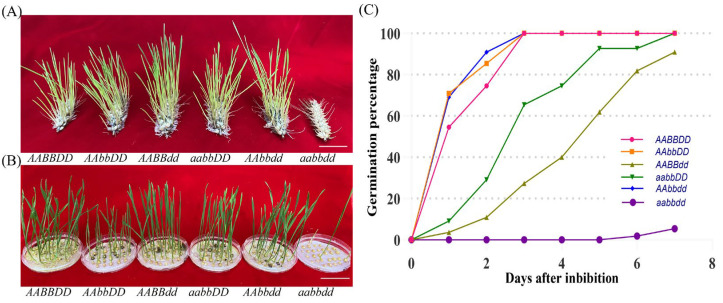
The triple homozygous mutant exhibited a significantly longer seed dormancy period. (**A**) Spike samples of different genotypes germinated in a dew chamber for 12 days. Scale bar, 3 cm. (**B**) Germination assays of different genotypes. Seeds were imbibed on petri dishes and germinated in a growth chamber at 23°C for 12 days. Scale bar, 5 cm. (**C**) Germination behaviors of 6 homozygous lines.

In the T_2_ generation derived from the T_0_ edited lines for *TaQsd1*, we selected five stable edited genotypes of *AAbbDD*, *AABBdd*, *aabbDD*, *AAbbdd*, and *aabbdd* for a systematic effect comparison test of various mutation combinations of different *TaQsd1* alleles on the PHS phenotype using their seeds at the physiologically mature stage. To more realistically simulate field PHS conditions, we simultaneously conducted whole-spike germination assays. Main stem and secondary tiller spikes of each edited line and the wild type were collected at physiological maturity and placed in a 23 °C constant-temperature incubator with spray misting to maintain humidity. The results showed that the spike germination rate of the *aabbdd* line was significantly lower than that of the wild type and other partial mutant types (*p* < 0.01). After 7 days of incubation, the germination rate of the wild type had exceeded 85% (87.3 ± 2.5%), while that of the *aabbdd* line was less than 10% (8.7 ± 1.8%); after 12 days of incubation, the germination rate of the *aabbdd* line remained below 30% (27.3 ± 3.2%), whereas all wild-type grains had already germinated (100 ± 0%). The remaining four partial knockout types (*AAbbDD*, *AABBdd*, *aabbDD*, and *AAbbdd*) showed no statistically significant difference in spike germination rate compared to the wild type, exhibiting a susceptible phenotype (Figure 3A–C).

**Figure 4:**
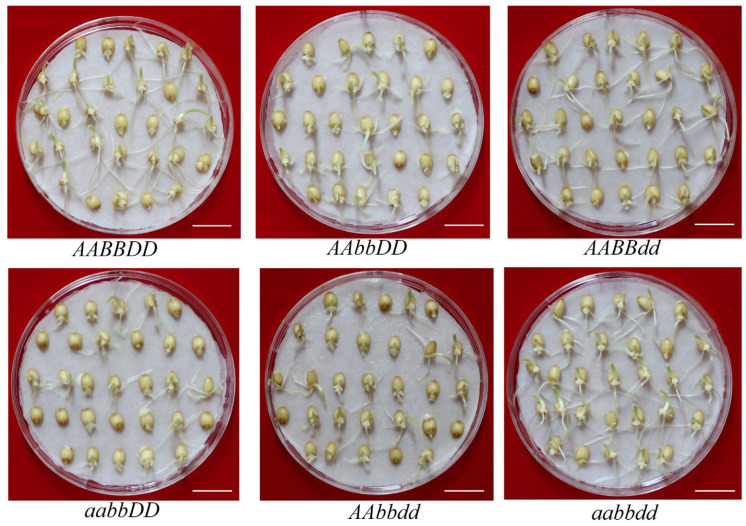
Seed germination assay at 30 days post-maturity. Scale bar, 1.5 cm.

The whole-spike germination results were highly consistent with the seed germination assay results. The results showed that only the mutation genotype *aabbdd*, in which all of the three homologous alleles of *TaQsd1* were completely knocked out, exhibited extremely strong PHS resistance: the time required for the seed germination rate reaching 50% was delayed by more than 7 days compared to the wild type (*AABBDD*), and its complete grain germination took over 30 days. It is noteworthy that, despite the significantly delayed germination process of the fresh harvest seeds of the *aabbdd* mutant, its seeds, maintained for one month after harvest, germinated normally, indicating an enhanced dormancy characteristic rather than a loss of germination ability of this mutation phenotype (Figure 4).

This test clearly demonstrates significant functional redundancy of the *TaQsd1* gene in regulating PHS, as only simultaneous inactivation of all three *TaQsd1* homologous genes could effectively increase seed dormancy and enhance sprouting resistance. To evaluate whether knockout of *TaQsd1* affects normal growth and development, we compared key agronomic traits of the edited lines with wild-type Lunxuan 13 at maturity. As shown in Table 3, no significant differences were observed between any of the edited lines and the wild type for plant height, spike length, spikelets per spike, grains per spike, or effective tillers per plant (*p* > 0.05). Notably, the triple homozygous mutant *aabbdd*, which exhibited significantly enhanced PHS resistance, showed normal agronomic performance comparable to the wild type, with plant height of 78.00 ± 0.00 cm, spike length of 7.00 ± 0.06 cm, spikelets per spike of 17.67 ± 0.33, grains per spike of 41.67 ± 0.88, and tillers per plant of 3.33 ± 0.33. These results indicate that loss of *TaQsd1* function does not compromise major agronomic traits at maturity.

## 3. Discussion

PHS is one of the most destructive abiotic stress disasters in global wheat production. When wheat encounters continuous rain or high humidity during the grain maturation stage, the kernels often germinate prematurely on the spike, leading to a decrease in thousand-kernel weight and test weight, resulting in yield losses and flour quality deterioration [32,33]. In the context of global climate change, extreme precipitation events are becoming more frequent and unpredictable, and the frequency and intensity of PHS continue to rise, making it a key bottleneck constraining the stable yield and quality of wheat [11]. Wheat resistance to PHS is a typical quantitative trait, co-regulated by multiple factors including seed dormancy depth, seed coat permeability, and hormone metabolism, particularly the balance between abscisic acid (ABA) and gibberellins (GA) [34,35,36,37,38]. Mining key genes conferring PHS resistance and breeding high-resistance varieties through genetic improvement have become a core strategy for ensuring global food security [11].

To date, several major genes associated with PHS resistance have been identified. Among them, the *R2R3-MYB*-type transcription factor family gene *TaMYB10* has been most extensively studied. This family comprises three homoeologous genes (*TaMYB10-A1*, *TaMYB10-B1*, *TaMYB10-D1*) located on chromosome 3, within the same genetic interval as the *R* locus controlling grain color [39]. The *TaMYB10* can activate genes in the flavonoid biosynthesis pathway, promoting anthocyanin accumulation and resulting in red grains; simultaneously, it can activate the NCED promoter, promoting ABA biosynthesis, thereby enhancing seed dormancy and improving PHS resistance [40,41,42,43]. However, the PHS resistance mediated by *TaMYB10-D1* is tightly linked to the red grain phenotype. Loss-of-function mutations (e.g., partial base deletions) lead to a change in grain color from red to white and reduce dormancy, making its direct application in white-grained wheat cultivars challenging [44,45]. 

Another class of core regulatory genes includes *TaPHS1* (located on chromosome 3A) and *MKK3* (located on chromosome 4A). *TaPHS1* encodes a protein homologous to phosphatidylethanolamine-binding protein (PEBP), while MKK3 encodes mitogen-activated protein kinase kinase 3. Both enhance PHS resistance by maintaining seed dormancy [46,47]. However, their expression is susceptible to temperature fluctuations, limiting their efficiency in breeding applications [46,48]. Furthermore, hormone signaling pathway-related genes such as *DOG1* (Delay of Germination 1) and *ABI5* (ABA Insensitive 5) also play crucial roles in PHS regulation. DOG1 influences dormancy depth by modulating the ABA/GA balance [49,50], while ABI5, as a core transcription factor downstream of ABA signaling, can activate multiple dormancy maintenance genes [51,52]. Nevertheless, the regulatory networks of these genes are complex. *DOG1* expression is developmentally regulated, and overexpression of *ABI5* may lead to reduced seedling emergence after sowing, affecting field establishment [52,53,54].

In contrast, the *Qsd* gene family has emerged as an ideal target for PHS improvement in white-grained wheat due to its unique advantages. The *Qsd1* was initially cloned in barley, mapped to chromosome 5H, and encodes alanine aminotransferase (AlaAT). Loss-of-function mutations of *Qsd1* can significantly prolong seed dormancy without affecting grain color or other agronomic traits [18]. Due to the high conservation between wheat and barley genomes, functionally conserved homoeologous genes of *TaQsd1* exist in the A, B, and D subgenomes of wheat. Abe et al. first used the CRISPR/Cas9 system to simultaneously knock out all three *TaQsd1* copies in the white-grained cultivar Fielder. The obtained mutants exhibited a 2–3 times longer seed dormancy period and significantly enhanced PHS resistance while maintaining the white grain phenotype and normal germination capacity [19].

In this study, through precise editing of the *TaQsd1* gene using CRISPR/Cas9 in the high-yielding, high-quality but PHS-susceptible white-grained wheat cultivar Lunxuan 13, we successfully generated edited lines with significantly improved PHS resistance. Under high-humidity conditions, the sprouting rate of spikes in these lines was reduced by over 60%, while their sowing germination rate remained above 90%. Furthermore, the original superior agronomic traits, such as high yield potential and lodging resistance, were preserved, achieving a synergistic optimization of resistance and practical utility for wheat production. It should be noted that in the present research, we neglected to specifically select the *Cas9*-free mutants in the T_2_ generation and just focused on the selection of genetically stable mutants at the *TaQsd1* loci, which is adverse to the possible field application of the edited lines. Despite the progress described above, future research needs to deepen in several directions: obtaining *Cas9*-free individuals in the segregation population of the hybrid cross between the edited mutants and their wild-type Lunxuan 13; elucidating the interaction network between *TaQsd1* and other dormancy regulatory genes; validating the field stability of resistance through multi-location and multi-year trials; clarifying its molecular mechanisms in regulating ABA/GA metabolism and amino acid homeostasis by integrating multi-omics technologies; and extending this editing strategy to other leading cultivars and exploring the synergistic effects of multi-gene editing. Overall, the *Qsd1* gene, with its advantages of functional conservation, stable resistance, and absence of undesirable linked phenotypes, has become a preferred target for the genetic improvement of PHS resistance in white-grained wheat. This work not only validates the application potential of the *Qsd1* gene but also improves the PHS resistance of the good wheat variety Lunxuan 13 for its revitalization.

## 4. Materials and Methods

### 4.1. Plant Materials and Bacterial Strains

The wheat variety Lunxuan 13 was kindly provided by Prof. Zhou Yang from the Institute of Crop Sciences, Chinese Academy of Agricultural Sciences, and it was cultivated in the experimental fields of the Chinese Academy of Agricultural Sciences (39° N, 116° E) under conventional field management practices, with a row spacing of 20 cm and a row length of 1.5 m, and irrigated monthly during the growth period. Immature seeds collected 15 days after flowering were used as material for genetic transformation experiments. The *Agrobacterium tumefaciens* strain GV3101 used in the experiments was purchased from Jiangsu CoWin Biotech Co., Ltd., Taizhou, China.

### 4.2. Gene Editing Vector Construction

Our laboratory had previously constructed the expression vector *pWMB110-Cas9*, on which the bar gene was used as a selectable marker, and the maize ubiquitin (ubi) promoter was employed to drive *Cas9* gene expression. Based on this, a single guide RNA (sgRNA) targeting the *TaQsd1* gene (5′-ACGGATCCACCTCCCTGCAG-3′) was designed, and the *TaU3* promoter was amplified using the genomic DNA from Lunxuan 13 as the template to regulate the expression of this sgRNA. Subsequently, the *TaU3-sgRNA* expression cassette was cloned into the *pWMB110-Cas9* vector, successfully constructing the recombinant plasmid *pWMB110-Cas9-TaQsd1*. Finally, the recombinant vector was introduced into the *Agrobacterium* GV3101 strain for subsequent genetic transformation experiments. The primers used for vector construction are listed in Supplementary Table S1.

### 4.3. Agrobacterium-Mediated Wheat Transformation

Immature seeds of Lunxuan 13 were collected from the field. Under sterile conditions, seed surface sterilization was performed through treatment with 75% ethanol for 1 min, followed by 10% sodium hypochlorite for 10 min, and finally rinsing three times with sterile distilled water. Immature embryos were isolated under a microscope, and transformation was carried out using an *Agrobacterium*-mediated genetic transformation method, specifically following the technical system established by Wang et al. [55]. Briefly, immature embryos were immersed in a suspension of *Agrobacterium* tumefaciens strain GV3101 harboring the binary vector *pWMB110-Cas9-TaQsd1*, prepared in WLS-inf liquid medium, for 10 min at room temperature. Following infection, the embryos were placed scutellum-side up onto WLS-AS co-cultivation medium and kept in darkness at 25 °C for 48 h. After co-cultivation, the embryonic axes were carefully excised with a sterile scalpel. The scutella explants were then transferred onto WLS-Res selection medium. After five days, the tissues were moved onto callus induction medium (WLS-P5). Following two weeks of culture, proliferating calli were subdivided and transferred to WLS-P10 medium for an additional three weeks to induce embryogenic callus formation. The embryogenic calli were subsequently transferred onto differentiation medium (LSZ-P5) and cultured at 25 °C under continuous light (100 μmol m^−2^ s^−1^) for 16 h every day. Regenerated shoots were excised and transferred onto MSF-P5 medium for further elongation and root development. Finally, well-rooted plantlets were transplanted into pots (20 × 30 cm) and acclimatized in a greenhouse under controlled conditions: 24 °C, a 16 h light/8 h dark photoperiod, 45% relative humidity, and a light intensity of 300 μmol m^−2^ s^−1^.

The obtained transgenic wheat plants were transplanted into 20 cm × 30 cm pots filled with conventional cultivation soil and grown in an artificial climate greenhouse. The growth conditions were set as follows: day/night temperature 24 ± 1 °C, photoperiod 16 h light/8 h dark, relative humidity 45 ± 5%, light intensity 300 μmol m^−2^ s^−1^ (supplemented by a lighting system), and aphid control using sticky colored boards (Zhengzhou Oukeqi Instruments Ltd., Zhengzhou, China). During growth, plants were watered weekly to maintain appropriate soil moisture. More detailed information on wheat cultivation and transformation procedures can be found in the study by Wang et al. [55].

### 4.4. Detection of Transgenic Wheat Plants

Genomic DNA was extracted from the young leaves of candidate transgenic wheat plants using the FastClean Plant Genomic DNA Kit (Jiangsu CoWin Biotech Co., Ltd., Taizhou, China). To identify positive transgenic plants, specific primers for the *bar* and *Cas9* genes (sequence information is provided in the Supplementary Materials) were designed. PCR amplification was performed using the 2× Rapid Taq Master Mix-P222 kit (Nanjing Vazyme Biotech Co., Ltd., Nanjing, China) on a Veriti 96-well gradient PCR instrument (Thermo Fisher Scientific, Waltham, MA, USA). The reaction program was set as follows: 95 °C pre-denaturation for 5 min, followed by 38 cycles (95 °C for 30 s, 60 °C for 20 s, and 72 °C for 1 min) and a final extension at 72 °C for 10 min. To detect the editing status of the three homologous *TaQsd1* genes, the target region was amplified using the PCR method described above. Edited plants were identified using a PCR-restriction enzyme (PCR-RE) assay: 5 μL of PCR product was taken, 0.3 U of *Pst*I (CTGCAG) restriction enzyme was added, and digestion was performed at 37 °C for 2 h in a 20 μL reaction volume. The digested products and undigested controls were separated by means of electrophoresis on a 1.5% agarose gel, and results were visualized using a GelDoc XR gel imaging system (Bio-Rad, Laboratories., Hercules, CA, USA). To precisely identify the mutation types of *TaQsd1* genes, PCR products from randomly selected mutant plants were sent for sequencing. The sequencing results were aligned with the *TaQsd1* gene sequences (*TraesCS5A02G216200*, *TraesCS5B02G214700*, and *TraesCS5D02G224200*) in the wheat reference genome database to determine specific gene editing types and mutation sites. The primers used for plant genotyping are listed in Supplementary Table S1.

### 4.5. Seed Dormancy Assay

Five healthy and plump seeds from each edited line with a mutation in either one of the three *TaQsd1-abd* alleles were carefully selected and sown individually in standard pots measuring 20 cm × 20 cm. The cultivation substrate consisted of sterilized conventional horticultural potting soil. Five biological replicates were set up for each line (one plant per pot). All plants were cultivated under identical environmental conditions (specific conditions as described in Section 2.3), with uniform water and fertilizer management and pest control measures. All naturally occurring tillers were retained during growth, but only seeds from the main spike and secondary spikes were ultimately collected for dormancy characterization. Seeds were harvested at 45 days after flowering (physiological maturity), with a total of 10 spikes harvested per line as experimental materials. For the assay, 30 uniformly sized grains were randomly selected from each main spike for the dormancy scoring experiment, in which wild-type Lunxuan 13 was set as the control. The specific procedure was as follows: each group of 30 seeds was evenly arranged in a Petri dish lined with two layers of filter paper, moistened with 3 mL of distilled water, and then placed in a 23 °C constant temperature incubator for dark incubation. Starting from the first day of incubation, germinated seeds were counted daily at a fixed time until germination ceased. Finally, the average germination rate (number of germinated seeds/total number of tested seeds × 100%) and its standard error for each line were calculated based on the cumulative germination data to assess differences in seed dormancy characteristics.

To further evaluate wheat PHS resistance under conditions simulating field humidity, a whole-spike germination assay was also performed. At physiological maturity (45 days after flowering), main stems and secondary tiller spikes were harvested from each edited line and wild-type Lunxuan 13 (10 spikes per line). The spikes were placed upright in a humidity chamber maintained at 23 °C with continuous spray misting to keep relative humidity above 90%. Germinated grains on each spike were counted daily at a fixed time for up to 12 days. For the spike germination assay, each line had five biological replicates (one spike per replicate). For each biological replicate, the germination rate was calculated based on three technical replicates, and the wild type was used as the control.

### 4.6. Seed Germination Assay at 30 Days Post-Maturity

Seed germination assays were conducted 30 days after the maturity of seeds from the main and secondary spikes. Wild-type Lunxuan 13 and the materials with mutations in the three *TaQsd1-abd* alleles were used, with 10 spikes harvested from each line as experimental materials. For the assay, 30 uniformly sized grains were randomly selected from each main spike for the germination experiment. The specific procedure was the same as in Section 4.5.

### 4.7. Statistical Analysis of Seed Dormancy

The SPSS 26.0 software package (SPSS, Chicago, IL, USA) was used for statistical analysis. One-way analysis of variance (ANOVA) followed by Tukey’s HSD post hoc test was used to compare germination rates among different lines. Data are presented as mean ± standard error (SE).

## Figures and Tables

**Table 1:** Transformation efficiency and editing efficiency of *TaQsd1* homologous genes in the T_0_ generation.

Item	Number	Efficiency (%)
Immature embryos transformed	78	–
T_0_ transgenic positive plants	41	52.6
T_0_ plants with targeted editing	27	65.9
*TaQsd1-5A* edited plants	21	51.2
*TaQsd1-5B* edited plants	23	56.1
*TaQsd1-5D* edited plants	17	41.5
Single-copy edited plants	7	25.9
Two-copy edited plants	6	22.2
Three-copy edited plants	14	51.9

**Table 2:** Inheritance of different knockout types for *TaQsd1* genes in T_1_ and T_2_ generations.

Line	T_1_ Generation	T_2_ Generation			Knockout Type (bp)
	*TaQsd1*-Edited Genotype	Plants Identified	*TaQsd1*-Edited Genotype	*p* Value (χ^2^ Test) *	
Ya33-1-3	*AABBDd*	23	6*AABBdd* + 12*AABBDd* + 5*AABBDD*	0.13	d: +1
Ya33-1-6	*AabbDD*	24	4*aaBBDD* + 15*AaBBDD* + 5*AABBDD*	1.58	a: −1 b: −11
Ya33-6-2	*AAbbDD*	17	17*AAbbDD*	–	b: −11
Ya33-17-4	*aabbdd*	19	19*aabbdd*	–	a: −1 b: −3 d: +1
Ya33-25-7	*AAbbDd*	21	5*AAbbdd* + 10*AAbbDd* + 6*AAbbDD*	0.14	b: −11 d: +1

* *p*-value greater than 0.05 indicates that the chi-square test supports the observed segregation ratio being consistent with the Mendelian inheritance ratio of 1:2:1.

**Table 3:** Agronomic traits of wild-type Lunxuan 13 and *TaQsd1*-knockout lines.

Genotype	Plant Height (cm)	Spike Length (cm)	Spikelets per Spike	Grains per Spike	Effective Tillers per Plant
*AABBDD*	78.00 ± 0.58	6.87 ± 0.03	16.33 ± 0.67	40.33 ± 0.88	3.33 ± 0.33
*AAbbDD*	78.67 ± 0.67	6.90 ± 0.06	17.00 ± 0.00	41.33 ± 1.20	2.67 ± 0.33
*AABBdd*	78.67 ± 0.33	6.83 ± 0.03	16.67 ± 0.33	39.00 ± 0.58	3.00 ± 0.00
*aabbDD*	77.67 ± 0.33	6.90 ± 0.06	17.33 ± 0.33	41.67 ± 2.03	3.33 ± 0.33
*AAbbdd*	79.67 ± 0.33	6.97 ± 0.07	16.67 ± 0.33	41.33 ± 0.67	4.00 ± 0.00
*aabbdd*	78.00 ± 0.00	7.00 ± 0.06	17.67 ± 0.33	41.67 ± 0.88	3.33 ± 0.33

No significant differences were detected between any edited line and wild-type Lunxuan 13 for all three traits (*p* > 0.05).

## Data Availability

The original contributions presented in this study are included in the article. Further inquiries can be directed to the corresponding authors.

## Supplementary Materials

The following supporting information can be downloaded at https://www.mdpi.com/article/10.3390/plants15091322/s1. Table S1: PCR primers for vector construction and identification of transgenic wheat plants; Table S2: Predicted and evaluated off-target sites of designed sgRNAs for the *TaQsd1* gene.
